# Vaccine Lymph from the Pustules Produced by Tartarized Antimony

**Published:** 1843-06

**Authors:** 


*Vaccine lymph from the pustules produced by Tartarized Antimony.*—In concluding the interesting and important subject of vaccination, and the consideration of the defences against small-pox, I may mention a singular statement made by Dr. Lichtenstein, and published in Hufeland's Journal for 1841. The paper in which this statement occurs is entitled, "On the Sources from which Matter preservative against the Small-pox has been derived," and the author asserts that limpid lymph taken from the pustules produced by tartarised antimony and inoculated in a person who has not been vaccinated, occasions a pock that cannot be distinguished from vaccinia. These pocks, he states, are equally protective against small-pox, and the lymph may be transmitted from person to person in the same manner with cow-pox. Dr. Lichtenstein inoculated and reinoculated thirty-one persons from this source, and none of these persons took the small-pox, although they mingled freely with the infected during the epidemic prevalence of small-pox.—*Wilson's Lectures* in *Lond. Lancet.*

